# Accuracy of direct genomic breeding values for nationally evaluated traits in US Limousin and Simmental beef cattle

**DOI:** 10.1186/1297-9686-44-38

**Published:** 2012-12-07

**Authors:** Mahdi Saatchi, Robert D Schnabel, Megan M Rolf, Jeremy F Taylor, Dorian J Garrick

**Affiliations:** 1 Department of Animal Science, Iowa State University, Ames, IA 50011, USA; 2 Division of Animal Sciences, University of Missouri, Columbia, MO 65211, USA; 3 Institute of Veterinary, Animal and Biomedical Sciences, Massey University, Palmerston North, New Zealand

## Abstract

**Background:**

In national evaluations, direct genomic breeding values can be considered as correlated traits to those for which phenotypes are available for traditional estimation of breeding values. For this purpose, estimates of the accuracy of direct genomic breeding values expressed as genetic correlations between traits and their respective direct genomic breeding values are required.

**Methods:**

We derived direct genomic breeding values for 2239 registered Limousin and 2703 registered Simmental beef cattle genotyped with either the Illumina BovineSNP50 BeadChip or the Illumina BovineHD BeadChip. For the 264 Simmental animals that were genotyped with the BovineHD BeadChip, genotypes for markers present on the BovineSNP50 BeadChip were extracted. Deregressed estimated breeding values were used as observations in weighted analyses that estimated marker effects to derive direct genomic breeding values for each breed. For each breed, genotyped individuals were clustered into five groups using K-means clustering, with the aim of increasing within-group and decreasing between-group pedigree relationships. Cross-validation was performed five times for each breed, using four groups for training and the fifth group for validation. For each trait, we then applied a weighted bivariate analysis of the direct genomic breeding values of genotyped animals from all five validation sets and their corresponding deregressed estimated breeding values to estimate variance and covariance components.

**Results:**

After minimizing relationships between training and validation groups, estimated genetic correlations between each trait and its direct genomic breeding values ranged from 0.39 to 0.76 in Limousin and from 0.29 to 0.65 in Simmental. The efficiency of selection based on direct genomic breeding values relative to selection based on parent average information ranged from 0.68 to 1.28 in genotyped Limousin and from 0.51 to 1.44 in genotyped Simmental animals. The efficiencies were higher for 323 non-genotyped young Simmental animals, born after January 2012, and ranged from 0.60 to 2.04.

**Conclusions:**

Direct genomic breeding values show promise for routine use by Limousin and Simmental breeders to improve the accuracy of predicted genetic merit of their animals at a young age and increase response to selection. Benefits from selecting on direct genomic breeding values are greater for breeders who use natural mating sires in their herds than for those who use artificial insemination sires. Producers with unregistered commercial Limousin and Simmental cattle could also benefit from being able to identify genetically superior animals in their herds, an opportunity that has in the past been limited to seed stock animals.

## Background

A variety of genotyping arrays, such as the BovineSNP50 BeadChip [1], can now be used to genotype cattle for at least 50 000 single nucleotide polymorphisms (SNP). The resultant SNP marker genotypes can be used to produce direct genomic breeding values (DGV) for selection candidates, as proposed by Meuwissen et al. [2], as soon as DNA can be obtained and without delaying selection to collect phenotypes. This allows reduced generation intervals, which might increase genetic progress [3]. First, SNP marker effects must be estimated from an analysis of a population with SNP genotypes and trait phenotypes (training set). The accuracies of the resulting DGV are key to the successful application of this new technology in genetic improvement. MacNeil et al. [4] showed that DGV could be considered as a correlated trait to that for which phenotypes are available for traditional estimation of breeding values. The square of the estimated genetic correlation represents the proportion of genetic variance explained by the genomic information and reflects the accuracy of the DGV. The objective of this study was to estimate genetic correlations between nationally evaluated traits and DGV in Limousin and Simmental beef cattle.

## Methods

### Genotype and phenotype data

A total of 2239 registered Limousin and 2703 registered Simmental animals were genotyped either at the University of Missouri (Columbia, MO) or by GeneSeek (Lincoln, NE). Most animals were genotyped with the BovineSNP50 BeadChip (Illumina, San Diego, CA) but 264 Simmental animals were genotyped with the BovineHD BeadChip (Illumina, San Diego, CA). The DNA for each animal was obtained from cryopreserved semen or hair samples provided by artificial insemination (AI) organizations or by numerous breeders of respective registered Limousin or Simmental cattle. For Limousin cattle, DNA was also available from samples sent to the University of Missouri for genetic testing for a mutation responsible for protoporphyria [5]. Birth year distributions for the genotyped animals within each breed are provided in Table 1.

**Table 1:** Birth year distribution for the genotyped Limousin and Simmental animals

**Birth year**	**Limousin**	**Simmental**
1966 to 1970	5	6
1971 to 1975	7	11
1976 to 1980	28	26
1981 to 1985	109	28
1986 to 1990	245	57
1991 to 1995	837	96
1996 to 2000	824	180
2001 to 2005	93	736
2006 to 2010	91	1563
Total	2239	2703

There are currently two versions of the BovineSNP50 BeadChip, referred to as B and C, which correspond to the initial product release and its re-synthesis, respectively. In this study, Simmental animals were genotyped with either version of the BovineSNP50 BeadChip or with the BovineHD BeadChip. All Limousin animals were genotyped with version B of the BovineSNP50 BeadChip. For animals that were genotyped with the BovineHD BeadChip, genotypes for markers that were common to versions B and C of the BovineSNP50 BeadChip were extracted. Since we assembled genotypes from different array versions for different breeds, which included monomorphic loci, the final genotype files included 56 794 markers for Limousin and 57 039 markers for Simmental. Only markers that passed quality control (call rate ≥ 0.95, minor allele frequency ≥ 0.005 and Hardy-Weinberg equilibrium test p-value > 1e-30) were used for further analyses. The genotype data were gathered from various sources (respective breed associations and various research projects) and some data was provided without call rate information. Consequently, the marker quality control tests were performed based on data from the 2896 Limousin or Limousin-Angus animals and the 1706 Simmental animals that were in the University of Missouri database at the time of analysis. The number of markers that passed all quality control tests and were used for analysis were 45 147 for Limousin and 47 364 for Simmental animals. Any missing genotypes (0.02% and 2.77% of all genotypes for the Limousin and Simmental animals, respectively) were replaced with the average value (on a 0–2 scale) for each SNP in that particular breed.

Deregressed estimated breeding values (DEBV), derived following Garrick et al. [6], were used as response variables to estimate SNP effects and their accuracies were used as weighting factors. This method results in DEBV that are free of parent average effects and the weights can be used to appropriately account for heterogeneous variance due to differences in reliabilities of individual and parent average EBV and therefore of corresponding DEBV. Ostersen et al. [7] confirmed in purebred pigs that using DEBV as the response variable yielded more accurate DGV than did using EBV. Expected progeny differences (EPD) and their Beef Improvement Federation (BIF) accuracies were obtained from each Breed Association for all of the genotyped animals, their sires and dams. The EPD were transformed to EBV by multiplying by 2 and the corresponding reliabilities (R^2^) were obtained as:

$$R^{2}=1-{\left(1-\text{BIF}\_\text{Accuracy}\right)}^{2}\text{.}$$

To derive the weighting factors, the proportion of genetic variance not explained by markers (parameter c of [6]) was assumed to be 0.40 and heritabilities that were used are in Table 2. In total, 15 traits were analyzed (some traits were recorded in only one breed, Table 2). The number of genotyped animals with DEBV varied among traits because some animals had no individual or offspring information contributing to their EPD. This occurred in some older animals born before the traits were introduced, and in some young genotyped animals. The number of genotyped animals with DEBV and the average DEBV reliabilities for the studied traits for each breed are in Table 2.

**Table 2:** Heritability, number of genotyped animals with DEBV and mean reliabilities of DEBV for studied traits

**Trait**		**Limousin**		**Simmental**	
	**h**^**2**^	**N**	**Reliability**	**N**	**Reliability**^**1**^
Birth weight	0.42	2185	0.65	2664	0.59
Calving ease direct	0.12	1687	0.61	2443	0.57
Calving ease maternal	0.13	1669	0.59	2441	0.43
Carcass weight	0.40	1459	0.67	2663	0.46
Docility	0.35	1225	0.54	-	-
Fat thickness	0.35	-	-	2463	0.59
Marbling	0.54	1447	0.61	2439	0.57
Rib eye muscle area	0.46	1449	0.62	2435	0.55
Scrotal circumference	0.43	1294	0.44	-	-
Shear force	0.40	-	-	1045	0.36
Stayability	0.21	757	0.50	563	0.66
Weaning weight direct	0.30	2150	0.53	2663	0.55
Weaning weight maternal	0.14	1480	0.55	2661	0.41
Yield grade	0.40	1446	0.62	2574	0.45
Yearling weight	0.29	1780	0.43	2663	0.56

Parent average contributions have been removed to calculate DEBV and their reliabilities. Heritabilities reported by North American Limousin Foundation and American Simmental Association.

### Statistical model

Method “BayesC” [8] was used to estimate marker effects for genomic prediction. This method assumes that non-zero SNP effects are drawn from a distribution with constant variance but that some known fraction of markers (π) have zero effect. Habier et al. [9] showed that BayesC is less sensitive to prior assumptions than BayesB. For each trait, the following model was fit to the DEBV data for training:

$$y_{i}=\mu +\overset{k}{\underset{j=1}{\sum }}z_{\mathrm{ij}}u_{j}+e_{i}\text{,}$$

where *y*_*i*_ is the DEBV for animal i, *μ* is the population mean, *k* is the number of marker loci in the panel, *z*_*ij*_ is allelic state (i.e., number of B alleles from the Illumina A/B calling system) at marker j in individual i, *u*_*j*_ is the random effect for marker j, with *u*_*j*_ ∼ *N*(0, *σ*_*u*_^2^) (with probability 1 - π) or *u*_*j*_ = 0 (with probability π), and *e*_*i*_ is a residual with heterogeneous variance, depending on the reliability of the DEBV information for animal i [6]. Parameter π was assumed to be 0.95 for all analyses. Markov chain Monte Carlo (MCMC) methods with 41 000 iterations were used to provide posterior mean estimates of marker effects and variances, after discarding the first 1000 samples for burn-in. In preliminary analyses, all genotyped animals were included in the training set (for each breed separately) to obtain estimates of genetic and residual variances for constructing priors for genetic and residual scale parameters.

The DGV for individual i within a validation set was derived as the sum over all k markers of posterior means of predicted SNP effects, as estimated in the training set, multiplied by the number of copies of the B allele:

$$DGV_{i}=\overset{k}{\underset{j=1}{\sum }}z_{\mathrm{ij}}{\hat{u}}_{j}$$

where DGV_i_ is the DGV for individual i in the validation dataset, *z*_*ij*_ is the marker genotype of individual i for marker j, and û_*j*_ is the posterior mean effect of marker j over the 40,000 post burn-in samples. All analyses were performed using GenSel software [10].

### K-means clustering and cross-validation

The accuracy of DGV was evaluated by pooling estimates from a 5-fold cross-validation strategy. For each breed, genotyped animals were first divided into five unequally-sized mutually exclusive groups. Each training analysis excluded one group when estimating marker effects, which were then used to predict DGV of individuals from the omitted group (validation set). This resulted in every animal having its predicted DGV obtained without considering its own DEBV.

K-means clustering was used to partition the genotyped animals into five groups within each breed, whereby relatedness was maximized within each group and minimized between each of the groups [11]. The CFC Package [12] was used to construct the numerator relationship matrix between genotyped animals for each breed, using pedigree information for genotyped animals and all their known ancestors, which comprised 13 790 Limousin and 16 716 Simmental individuals. The Hartigan and Wong [13] algorithm, implemented using R [14], was used for K-means clustering based on a difference matrix obtained from the numerator relationship matrix among the genotyped animals. The maximum genetic relationship coefficient (a_max_) was calculated between each animal and all other animals within the same group, and between each animal and all other animals in the other four groups, so that each animal had two a_max_ values (within and between groups). The average a_max_ values of animals of each group were calculated within and between groups to quantify the quality of the clustering. Further details are in Saatchi et al. [11].

### Genetic correlations between trait and its DGV

We applied a weighted bivariate animal model using the DGV, computed as described above for each genotyped animal, and their DEBV to estimate variance and covariance components for each of the studied traits in each breed. The purpose of fitting this model was to estimate the genetic correlation between the trait (T) and its respective DGV (*r*_*g*(*T*,*DGV*)_). This trait-DGV genetic correlation is required to pool DGV and traditional EBV in national genetic evaluation [4]. The model was:

$$\left[\begin{array}{c} \text{DEBV}\\ \text{DGV} \end{array}\right]=\left[\begin{array}{c} \begin{array}{cc} X_{1} & 0 \end{array}\\ \begin{array}{cc} 0 & X_{2} \end{array} \end{array}\right]\left[\begin{array}{c} \beta_{1}\\ \beta_{2} \end{array}\right]+\left[\begin{array}{cc} Z_{1} & 0\\ 0 & Z_{2} \end{array}\right]\left[\begin{array}{c} \alpha_{1}\\ \alpha_{2} \end{array}\right]+\left[\begin{array}{c} e_{1}\\ e_{2} \end{array}\right]\text{,}$$

where ***β***_1_ and ***β***_2_, are vectors of fixed effects (only the trait mean for ***β***_1_ but class effects of the five groups for ***β***_2_); ***α***_1_ and ***α***_2_, are vectors of random additive genetic effects for the trait and its DGV, respectively, where $Var\left(\alpha_{1}\right)=G\sigma_{\alpha_{1}}^{2}$, $Var\left(\alpha_{2}\right)=G\sigma_{\alpha_{2}}^{2}$ and $Cov\left(\alpha_{1},\alpha_{2}\right)=G\sigma_{\alpha_{1}\alpha_{2}}$, where $\sigma_{\alpha_{1}}^{2}$ is the additive genetic variance of trait, $\sigma_{\alpha_{2}}^{2}$ is the additive genetic variance of DGV ($\sigma_{\alpha_{2}}^{2}/\sigma_{\alpha_{1}}^{2}$ represents the proportion of additive genetic variance of a trait explained by markers) and $\sigma_{\alpha_{1}\alpha_{2}}$ is the genetic covariance between trait and its DGV; **G** consists of nonzero elements of **A**, the usual pedigree-based numerator relationship matrix among individuals in the same group, but with covariances between individuals in different groups zeroed out as follows:

$$G=\left[\begin{array}{l} \begin{array}{cc} A_{11} & \begin{array}{ccc} 0 & 0 & \begin{array}{cc} 0 & 0 \end{array} \end{array} \end{array}\\ \begin{array}{cc} 0 & \begin{array}{ccc} A_{22} & 0 & \begin{array}{cc} 0 & 0 \end{array} \end{array} \end{array}\\ \begin{array}{cc} 0 & \begin{array}{ccc} 0 & A_{33} & \begin{array}{cc} 0 & 0 \end{array} \end{array} \end{array}\\ \begin{array}{cc} 0 & \begin{array}{ccc} 0 & 0 & \begin{array}{cc} A_{44} & 0 \end{array} \end{array} \end{array}\\ \begin{array}{cc} 0 & \begin{array}{ccc} 0 & 0 & \begin{array}{cc} 0 & A_{55} \end{array} \end{array} \end{array} \end{array}\right]\text{.}$$

This approach effectively pools information across all five groups to estimate the genetic parameters. The remaining effects **e**_**1**_ and **e**_**2**_ are vectors of mutually uncorrelated random residual effects for the two traits, $\text{Var}\left(e_{1}\right)=I\sigma_{e_{1}}^{2}$ and $\text{Var}\left(e_{2}\right)=W\sigma_{e_{2}}^{2}$, cov(***e***_1_, ***e***_2_) = **0**, where ***I*** is an identity matrix and ***W*** is a diagonal matrix containing weights (the same weights as used in the estimation of SNP effects) based on the reliability of the corresponding DEBV [6]; ***X***_***i***_ and ***Z***_***i***_ are known design matrices for fixed effects and random additive genetic effects, respectively. Variance components were estimated by restricted maximum likelihood (REML) using the ASReml v3.0 software package [15].

To evaluate the efficiency of selection based on DGV in relation to selection based on parent average information, relative selection responses were computed for each trait as the ratio of two accuracies [16]:

$$\text{Efficiency}=\frac{\text{Accuracy}\;of\;\text{DGV}}{\text{Accuracy}\;of\;PA}=\frac{r_{g\left(T,\text{DGV}\right)}}{\sqrt{R_{\mathrm{PA}}^{2}}}$$

where *r*_*g*(*T*,*DGV*)_ is the genetic correlation between the trait and its DGV, and *R*_*PA*_^2^ is the reliability of parent average information. The available parental information in our dataset represents more than that available on the parents of the genotyped bulls at the time of their birth because they include information on progeny and grandprogeny that would not normally exist at the time of selection. In this efficiency formula, the selection intensity and generation interval were assumed identical for the two selection strategies, which is true when DGV information is available at the same time as parent average information.

The efficiency of selection on DGV versus parent average was also computed for the most recent crop of calves - those non-genotyped purebred Simmental animals that were born after January 2012 (overall 323 animals). EPD and BIF accuracies of their sires and dams were obtained from the American Simmental Association evaluation in October 2012.

## Results

### K-means clustering

The number of individuals and average a_max_ within and between the K-means clustered groups for Limousin and Simmental animals are presented in Table 3. The average a_max_ was much larger within groups than between groups. These results show that the K-means clustering successfully partitioned individuals into related groups with reduced relationships between groups.

**Table 3:** The number of individuals and the averages (± standard deviation)

	**Limousin**				
**Group**	**1**	**2**	**3**	**4**	**5**
Number	638	376	695	182	348
a_max_ within	0.43±0.12	0.47±0.10	0.38±0.15	0.48±0.09	0.40±0.13
a_max_ between	0.23±0.11	0.29±0.15	0.28±0.15	0.27±0.16	0.27±0.15
	**Simmental**				
**Group**	**1**	**2**	**3**	**4**	**5**
Number	606	876	220	876	125
a_max_ within	0.52±0.08	0.35±0.12	0.28±0.04	0.34±0.13	0.50±0.06
a_max_ between	0.17±0.13	0.22±0.19	0.25±0.18	0.25±0.18	0.24±0.18

The average of the maximum genetic relationship coefficient (a_max_) within and between groups for five groups formed by K-means clustering in Limousin and Simmental animals.

### Genetic correlations between traits and DGV

The estimated genetic (co) variances of each trait and its DGV are shown in Table 4 and the estimated heritabilities of DGV and the estimated trait-DGV genetic correlations are shown in Table 5. For most traits, estimates of genetic variance of DGV were smaller than the corresponding estimates of additive genetic variance and were almost the same as the estimates of genetic covariance between traits and their respective DGV. Heritabilities of DGV were 1.00 for most traits. In Limousin animals, estimates of the genetic correlation of the trait with its DGV were higher than 0.40 for all traits except for stayability (0.39±0.06). For Simmental animals, estimates of the genetic correlation of the trait with its DGV were higher than 0.30 for all traits except fat thickness (0.29±0.02). Estimates of the genetic correlation of the trait with its DGV were higher than 0.50 for birth weight, carcass weight, marbling, rib eye muscle area, weaning weight direct and yield grade, in both breeds.

**Table 4:** Estimates of genetic (co)variances of traits and their respective DGV for Limousin and Simmental animals

**Trait**	**Limousin**			**Simmental**		
	$\sigma_{\alpha_{1}}^{2}$	$\sigma_{\alpha_{2}}^{2}$	$\sigma_{\alpha_{1}\alpha_{2}}$	$\sigma_{\alpha_{1}}^{2}$	$\sigma_{\alpha_{2}}^{2}$	$\sigma_{\alpha_{1}\alpha_{2}}$
Birth weight (kg)	2.80	0.93	0.93	3.49	1.29	1.39
Calving ease direct (%)	55.92	11.46	13.12	108.62	29.90	25.72
Calving ease maternal (%)	78.12	25.91	22.97	118.60	28.24	18.50
Carcass weight (kg)	107.82	25.67	29.67	122.07	30.87	36.49
Docility (%)	423.76	84.86	75.85	-	-	-
Fat thickness (mm)	-	-	-	7.10	1.29	0.64
Marbling (units)	0.009	0.002	0.003	0.145	0.051	0.054
Rib eye muscle area (cm2)	5.00	1.53	1.73	12.82	4.16	4.33
Scrotal circumference (mm)	831.28	154.71	162.65	-	-	-
Shear force (kg)	-	-	-	0.085	0.021	0.022
Stayability (%)	93.07	11.91	12.96	114.30	28.00	32.90
Weaning weight direct (kg)	68.08	18.76	20.80	75.14	18.01	19.13
Weaning weight maternal (kg)	26.00	2.83	3.94	53.37	10.14	7.86
Yield grade (units)	0.018	0.005	0.006	0.073	0.026	0.027
Yearling weight (kg)	111.76	22.82	38.44	726.42	61.77	96.28

$\sigma_{\alpha_{1}}^{2}$ is the additive genetic variance of trait, $\sigma_{\alpha_{2}}^{2}$ is the additive genetic variance of DGV as a proportion of trait additive genetic variance) and $\sigma_{\alpha_{1}\alpha_{2}}$ is the genetic covariance between the trait and its DGV.

**Table 5:** Estimates of heritability of DGV and of genetic correlations between trait and its DGV

**Trait**	**Limousin**		**Simmental**	
	***h***_***DGV***_^**2**^	***r***_***g*(*T*,*DGV*)**_	***h***_***DGV***_^**2**^	***r***_***g*(*T*,*DGV*)**_
Birth weight	1.00±0.00	0.58±0.04	1.00±0.00	0.65±0.03
Calving ease direct	1.00±0.00	0.52±0.05	1.00±0.00	0.45±0.02
Calving ease maternal	1.00±0.00	0.51±0.03	0.99±0.02	0.32±0.02
Carcass weight	1.00±0.00	0.56±0.06	1.00±0.00	0.59±0.04
Docility	1.00±0.00	0.40±0.04	-	-
Fat thickness	-	-	0.98±0.02	0.29±0.02
Marbling	1.00±0.00	0.65±0.06	1.00±0.00	0.63±0.04
Rib eye muscle area	1.00±0.00	0.63±0.05	1.00±0.00	0.59±0.04
Scrotal circumference	1.00±0.00	0.45±0.05	-	-
Shear force	-	-	1.00±0.00	0.53±0.08
Stayability	1.00±0.00	0.39±0.06	1.00±0.00	0.58±0.06
Weaning weight direct	1.00±0.00	0.58±0.04	1.00±0.00	0.52±0.04
Weaning weight maternal	1.00±0.00	0.46±0.07	1.00±0.00	0.34±0.03
Yield grade	1.00±0.00	0.67±0.05	1.00±0.00	0.62±0.06
Yearling weight	1.00±0.00	0.76±0.08	1.00±0.00	0.45±0.02

Heritability of DGV (*h*_*DGV*_^2^ ± *SE*) and genetic correlations between trait and its DGV (*r*_*g*(*T*,*DGV*)_±SE) estimated from bivariate animal models in Limousin and Simmental.

### Efficiencies of selection on DGV versus parent average

Table 6 presents the efficiencies of selection based on DGV in comparison to selection based on parent average information for genotyped animals in both breeds. Efficiencies differed between traits and ranged from 0.68 (docility) to 1.28 (yearling weight) in Limousin, and from 0.51 (fat thickness) to 1.44 (shear force) in Simmental. Efficiency values larger than 1 (meaning that indirect selection based on DGV is expected to produce greater genetic response than direct selection based on parent average information) were observed for marbling, rib eye muscle area, yield grade and yearling weight traits in Limousin, and for birth weight, carcass weight, marbling, rib eye muscle area, shear force and yield grade traits in Simmental.

**Table 6:** The reliabilities of parent average and the efficiency of selection on DGV versus parent average

**Trait**	**Limousin (Genotyped animals)**		**Simmental (Genotyped animals)**		**Simmental (Young animals)**^**1**^	
	***R***_***PA***_^**2**^	**Efficiency**	***R***_***PA***_^**2**^	**Efficiency**	***R***_***PA***_^**2**^	**Efficiency**
Birth weight	0.42	0.89	0.37	1.06	0.37	1.08
Calving ease direct	0.41	0.81	0.36	0.75	0.33	0.78
Calving ease maternal	0.41	0.80	0.34	0.55	0.28	0.60
Carcass weight	0.39	0.89	0.31	1.07	0.28	1.11
Docility	0.35	0.68	-	-	-	-
Fat thickness	-	-	0.33	0.51	0.22	0.62
Marbling	0.38	1.05	0.33	1.10	0.20	1.41
Rib eye muscle area	0.38	1.02	0.32	1.05	0.19	1.35
Scrotal circumference	0.32	0.80	-	-	-	-
Shear force	-	-	0.14	1.44	0.07	2.04
Stayability	0.32	0.69	0.17	1.39	0.15	1.48
Weaning weight direct	0.40	0.92	0.35	0.87	0.33	0.91
Weaning weight maternal	0.39	0.74	0.35	0.57	0.28	0.64
Yield grade	0.38	1.08	0.28	1.17	0.20	1.40
Yearling weight	0.36	1.28	0.36	0.75	0.32	0.79

^1^Non-genotyped purebred Simmental animals that were born after January 2012 (overall 323 animals).

Table 6 also presents the reliabilities of parent averages and the efficiencies of selection on DGV versus parent average for non-genotyped young Simmental animals. In general, reliabilities of parent average were lower for non-genotyped young animals than those calculated for genotyped Simmental animals. Efficiencies are influenced by the reliabilities of parent average. Higher efficiencies were obtained for non-genotyped young animals, ranging from 0.60 (calving ease maternal) to 2.04 (shear force) over all studied traits. The results show that parent average reliabilities are quite variable, ranging from zero to 0.50, and show benefits for selection on DGV versus parent average (efficiency larger than 1) for some Simmental animals even for traits with overall efficiency smaller than 1 (Additional file 1). Some traits exhibit bimodal distributions of parent average reliabilities, reflecting the difference in reliabilities between animals sired by natural mating compared to artificial insemination (AI) sires.

## Discussion

The accuracy of DGV cannot be assessed in the training set but must be assessed in a sample of individuals that are not included in training. Multi-fold cross-validation in beef cattle has some advantages described below in comparison to partitioning the genotyped animals into two groups (old and young animals), with training in older animals and validation in younger animals, which is the usual approach in dairy cattle studies [17,18]. Using multi-fold cross-validation, the DGV can be obtained for all genotyped animals in validation sets, while large training sets can be retained.

Habier et al. [19] showed that the accuracies of DGV depend on both genetic relationships between individuals in the training and validation sets and on linkage disequilibrium (LD) between markers and quantitative trait loci (QTL). They showed that the accuracy of DGV for a selection candidate decreases as the average genetic relationship of the candidate to the training set individuals decreases. In beef cattle, many registered selection candidates are produced by natural mating sires, which may be distantly related to the individuals in training sets. If the accuracies of DGV are more dependent on genetic relationships than on marker-QTL LD, then the effectiveness of genomic selection will be limited in practice for such distantly-related selection candidates. Saatchi et al. [11] showed that conservative accuracies of DGV that are less affected by relationships can be obtained by minimizing the genetic relationships between training and validation sets using K-means clustering. In this study, we also used K-means clustering and found greater a_max_ values between groups than reported by Saatchi et al. [11] for Angus beef cattle. This indicates that the genetic relationships were greater between training and validation sets for the Limousin and Simmental populations used here than for the Angus population used by Saatchi et al. [11].

In simulation studies, the correlation between DGV and true breeding values (TBV) has been used to represent the accuracy of DGV. However, in field data, TBV are not available and the correlation between DGV and the response variable (phenotype records, EBV, DEBV, etc.) is commonly used to derive the accuracy of DGV. In dairy cattle, the correlation between DGV and DEBV (or EBV) is a good estimate of the accuracy of DGV, because the reliabilities of DEBV are high. However, in beef cattle, for which the reliabilities of DEBV are usually low (less than 0.70, Table 2), these correlations typically underestimate the accuracy of DGV due to the contribution of environmental effects and random error to the DEBV. In some studies, the correlation between the DGV and the response variable is divided by the square root of the average reliability of the response variable [17,20] to adjust for the underestimation of the accuracy. However, this adjustment does not consider the heterogeneous error variance that is associated with DEBV when they have different reliabilities in the validation animals, which may lead to bias. Saatchi et al. [11] standardized the covariance between DGV and DEBV by an estimate of the genetic variance to estimate the accuracy of DGV in American Angus beef cattle. In this study, estimates of genetic variance were not available to apply that method. Instead, the estimate of the genetic correlation of a trait with its DGV was used to estimate the accuracy of DGV, as the square of these correlations represents the proportion of genetic variance accounted for by the genomic information if the DGV has heritability 1.

In general, the DGV accuracies obtained here are lower than those reported for dairy cattle for traits with similar heritabilities [17,18,21]. Saatchi et al. [11] also reported that accuracies of DGV using the BovineSNP50 BeadChip were lower in Angus beef cattle than in dairy cattle. One reason is that the accuracies of EBV (used to derive the DEBV response variable) are lower in beef cattle than in dairy cattle because of a less extensive use of artificial insemination in beef bulls having fewer progeny with production records [22,23]. Estimates of SNP effects and resulting DGV will be more accurate as the accuracies of EBV (or DEBV) increase, because the response variable will be closer to the TBV. In dairy cattle, the accuracies of DEBV in the training set are much higher than in beef cattle and the number of animals with high accuracy DEBV is greater. The average accuracy of the EBV for traits studied by Su et al. [21] was 0.89, compared to 0.57 and 0.52 in our Limousin and Simmental populations. The size of training population is an important factor affecting the accuracies of DGV [2], which is typically higher in dairy than beef cattle [17,18,21]. Furthermore, it has been common for dairy cattle studies to validate DGV on progeny [17], and progeny are more highly related to the training population than the situation we have created here with K-means clustering. Also, different extents and patterns of LD have been reported for beef and dairy cattle [24,25], which could contribute to the lower accuracy of DGV reported here. The different approaches used to measure the DGV accuracies could also explain these differences.

Estimates of variances and of covariances between traits and their respective DGV obtained in this study indicate that the heritabilities of the DGV were 1 for most traits in both the Limousin and Simmental population. Heritabilities of 1 are expected for perfectly inherited attributes, such as SNP genotypes or linear functions of SNP genotypes. However, heritabilities less than 1 (ranging from 0.75 to 0.95) were estimated in Angus cattle using a similar K-means clustering and cross-validation approach [11]. In that study, the complete numerator relationship matrix among individuals of all clustered groups was used in the bivariate animal model, rather than a matrix with zero covariances between animals that are in different groups. By using the full relationship matrix, the heritability of the DGV was underestimated in [11] because the linear functions that predict DGV were different for each group. Using the complete numerator relationship matrix also resulted in estimates of the trait heritability from bivariate analyses to be biased downwards ([11]) compared to the values used in national evaluations ([11], Table 2). This downwards bias was removed when the DEBV were used to estimate heritability in a single trait model, i.e. ignoring the correlated DGV. Setting the relationships between animals in different groups to zero results in the derivative of the likelihood function being pooled from the derivatives of the likelihood functions that would be obtained from separate analysis of each group. Furthermore, when setting relationships between groups to zero, the heritability of the DGV is depressed only by SNP genotyping errors and the heritability of the DEBV is essentially the same as that obtained from single trait analyses of DEBV. Zeroing relationships between groups results in a block structure to its inverse of the variance-covariance matrix and avoids any cross-products between DEBV used to derive an individual’s DGV and the individual’s DGV. That cross-product introduces some residual covariance between DEBV and DGV but these are assumed zero in the bivariate model used here. We believe that this approach makes better use of the data than could be achieved by estimating the genetic correlation between DEBV and DGV separately in each group and then pooling those estimates.

The estimated trait-DGV genetic correlations varied between traits due to different quantities of information and possibly different genetic architectures between the traits (Table 5). Hayes et al. [26] showed that the accuracy of genomic predictions is higher for traits with a higher proportion of large effect loci than for traits with no loci of large effect. Furthermore, the LD between BovineSNP50 BeadChip loci and QTL could differ between traits and between breeds. The difference in the trait-DGV genetic correlations was relatively small between low and high heritability traits due to the use of DEBV as the response variable, which makes accuracies less dependent on heritability itself and more a function of the EBV accuracies. In general, estimates of trait-DGV genetic correlations were higher in Limousin than in Simmental animals (the averages across traits were 0.55 and 0.50 for Limousin and Simmental, respectively). This may be because registered Limousin animals have a more homogeneous genetic background than the Simmental animals, as the a_max_ values between groups were higher for Limousin than for Simmental. Both these US associations allow registration of crossbreds with other beef cattle breeds but upgrading and composite cattle are more common for the American Simmental Association than for the North American Limousin Foundation.

The estimated trait-DGV genetic correlations reported here for Limousin and Simmental animals were lower than those reported for Angus beef cattle by Saatchi et al. [11] for most traits that were in common in these studies. This could be due to the different selection strategies practiced within Angus compared to Continental breeds or due to differing allele frequencies among the breeds, which could affect the extent of LD between markers and causative genes and consequently the accuracies of DGV. Also, larger training population sizes (about 3570 total genotyped animals) were used in Angus [11]. Using 1006 Angus animals genotyped with a proprietary 384 SNP panel developed by Igenity (Duluth, GA), MacNeil et al. [4] reported a lower trait-DGV genetic correlation for marbling (0.38) than obtained here in Limousin and Simmental animals (correlations of 0.65 or 0.63, respectively, Table 5). The different SNP panels used for the prediction of DGV and different training and validation populations largely explain these differences. In the commercial implementation of genomic prediction in these breeds, training will use all genotyped animals from each breed to predict DGV for selection candidates, which usually are young animals without phenotype records. Thus, higher correlations than reported here are expected due to the larger training dataset sizes (from all genotyped animals) and closer genetic relationships between training and implementation populations.

In beef cattle, birth weight is typically the only observation on a young bull at the time of castration when a decision is made to retain the bull. While the animal’s birth weight may contribute to EPD calculated for weaning weight direct and yearling weight in a multi-trait analysis performed before selection, in general, parent average information is the main source of information available for selecting young animals. The efficiencies of selection based on DGV in comparison to selection based on parent average information (Table 6) indicate that selection on DGV was more efficient than using parent average information only for some traits. In general, the parent averages used here have higher reliabilities than parent averages that are available at the time of birth of their progeny because they include information on progeny and grandprogeny that would not normally exist at the time of selection. Also, the available parent averages are not adjusted for selection to account for the Bulmer effect. Bijma [27] showed that the accuracy of parent average is dramatically reduced by selection, up to a factor of three fold, which is ignored when computing reliabilities in national genetic evaluations. This leads to an underestimation of the efficiency of selection on DGV relative to selection on parent average when the DGV accuracies obtained by cross-validation are compared to parent average accuracies obtained from national genetic evaluation.

Reliabilities of parent average are lower and so the efficiencies of selection on DGV versus parent average are higher for non-genotyped young animals, which represent the selection candidates. The distribution of parent average reliabilities exhibits considerable variation within and between traits and therefore there is considerable variation in efficiencies of selection on DGV versus parent average (see Additional file 1). Variation in parent average reliabilities within a trait reflects the difference in reliabilities between natural mating sires that have few progeny and AI sires that can have many progeny. Unlike dairy cattle, natural mating is widely used in the beef industry. These factors lead to bimodal distributions of parent average reliabilities for some traits. Even for traits with efficiencies smaller than 1, there are opportunities for selection on DGV at least for that fraction of the population sired by natural mating sires. The proportions of registrations from natural mating sires are about 63% among Limousin calves in 2011 (Bob Weaber, K-State University, personal communication) and about 40% among Simmental calves (Wade Shafer, American Simmental Association, personal communication). This means that there are more benefits from selecting on DGV for breeders who use natural mating sires in their herds.

Here, we deliberately tried to minimize the relationship between training and validation set individuals by K-means clustering to establish lower bounds for prediction accuracies. In practice, training will be performed on all genotyped animals and predictions will be implemented in young animals (creating higher genetic relationships between training and validation or implementation sets) and so even higher efficiencies for selecting on DGV are expected. However, parent average information (and/or the animal’s own records) could be blended with DGV information for selecting young animals, for example using a selection index approach [17]. Saatchi et al. [11] combined the DGV and adjusted parent average information in a selection index for 16 traits in Angus beef cattle and showed that the accuracies of blended information are equal or a little higher than the accuracies of the most informative source of information (either DGV or parent average).

Progeny testing is a strategy that is commonly used to increase the accuracy of the predicted genetic merit of selection candidates but it increases generation intervals from 2.5 years when using DGV to about 5.5 years [28]. Furthermore, progeny testing increases the cost of breeding operations and in cattle is practically limited only to males, which potentially can have many progeny, while DGV can be obtained for females with the same accuracies as for males. The availability of DGV creates new opportunities for both commercial Limousin and Simmental producers to identify superior animals in their herds. To date, this opportunity has been limited to seed stock animals enrolled in performance-recording programs. The beef industry will continue to need to record phenotypes to retrain genomic predictions for which accuracies will otherwise erode in successive generations [20,29].

## Conclusions

This study applied genomic prediction to US Limousin and Simmental beef cattle. By minimizing the genetic relationships between training and validation groups using K-means clustering, lower bounds for the accuracy of genomic predictions were established based on the genetic correlation between the trait and its DGV. Estimates ranged from 0.39 to 0.76 in Limousin and from 0.29 to 0.65 in Simmental cattle. The efficiency of selection based on DGV in comparison to selection based on parent average information for genotyped animals ranged from 0.68 to 1.28 in Limousin and from 0.51 to 1.44 in Simmental cattle. However, these efficiencies are likely to be significantly underestimated because estimates of the accuracies of parent averages used in this retrospective study are higher than those that are generally available at the birth of selection candidates and are higher than those adjusted for the effects of selection. The reliabilities of parent average were lower for non-genotyped young animals, and the efficiencies of selection on DGV versus parent average were higher and ranged from 0.60 to 2.04 in Simmental calves. These results demonstrate the feasibility of implementing DGV for Limousin and Simmental beef cattle and indicate that greater genetic responses can be achieved through the use of DGV in comparison to parent average information for at least some traits in that fraction of the population sired by natural mating. Both Limousin and Simmental breeders will benefit from using DGV information to increase genetic progress in their populations.

## Competing interests

The authors declare that they have no competing interests.

## Authors' contributions

MS carried out the study, performed the statistical analyses and drafted the manuscript. RDS carried out quality controls for markers. MMR extracted the DNA for some Limousin animals. DJG conceived the study, and participated in its design and coordination. DJG, JFT and RDS critically contributed to the final version of manuscript. All authors read and approved the final manuscript.

## Supplementary Material

Additional file 1 **The distribution of parent average reliabilities for all studied traits in non-genotyped young Simmental animals. **Non-genotyped purebred Simmental animals that were born after January 2012 (overall 323 animals). Click here for file
