# Supplementary material for: Accuracy of direct genomic breeding values for nationally evaluated traits in US Limousin and Simmental beef cattle
Source: Genet Sel Evol. 2012 Dec 7;44(1):38. doi: 10.1186/1297-9686-44-38 (PMC3536607; doi:10.1186/1297-9686-44-38)
Supplement: Additional file 1 — The distribution of parent average reliabilities for all studied traits in non-genotyped young Simmental animals. Non-genotyped purebred Simmental animals that were born after January 2012 (overall 323 animals). [file 1297-9686-44-38-S1.pdf]

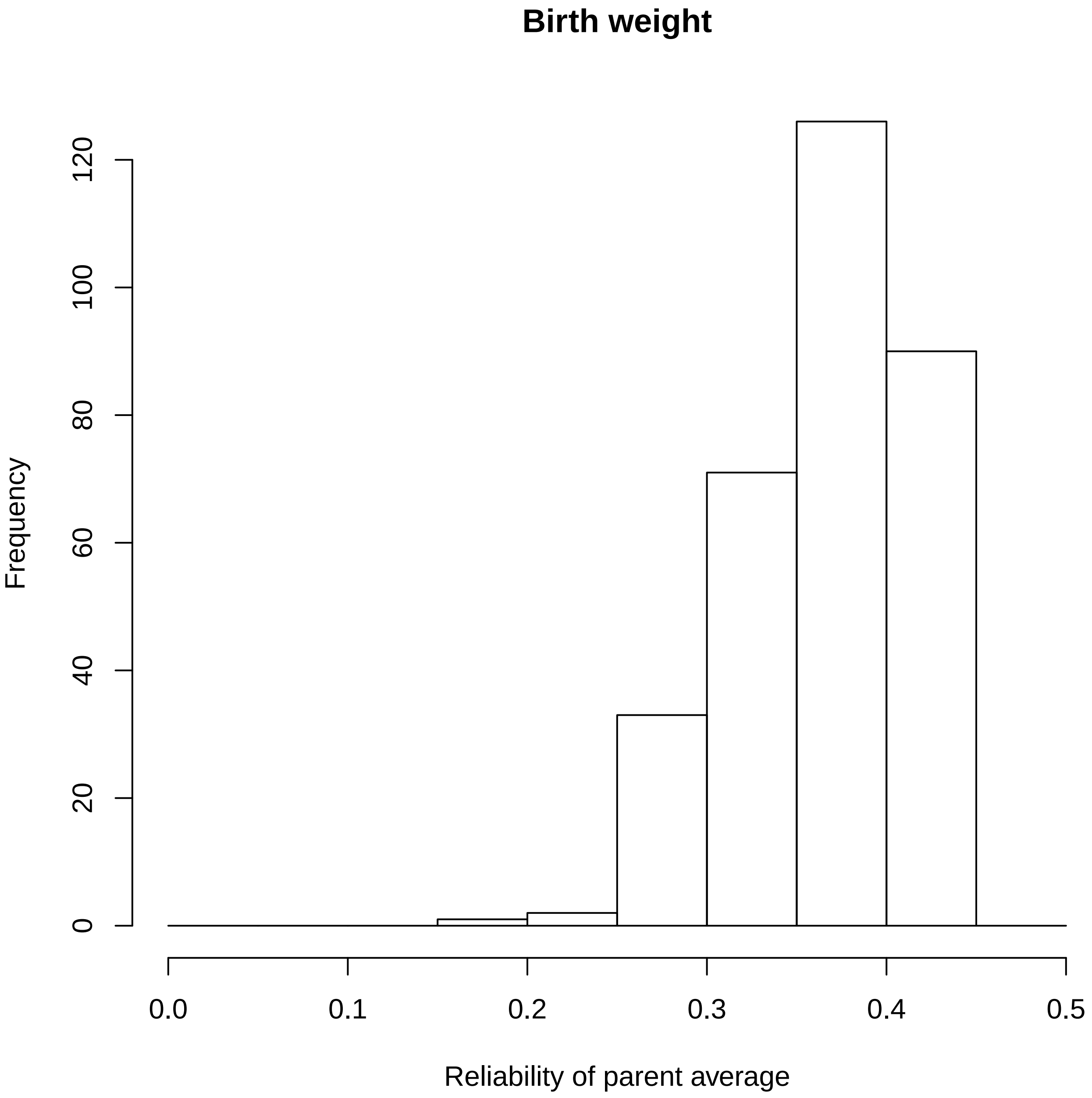
Birth weight
Frequency
0
20
40
60
80
100
120
0.0
0.1
0.2
0.3
0.4
0.5
Reliability of parent average

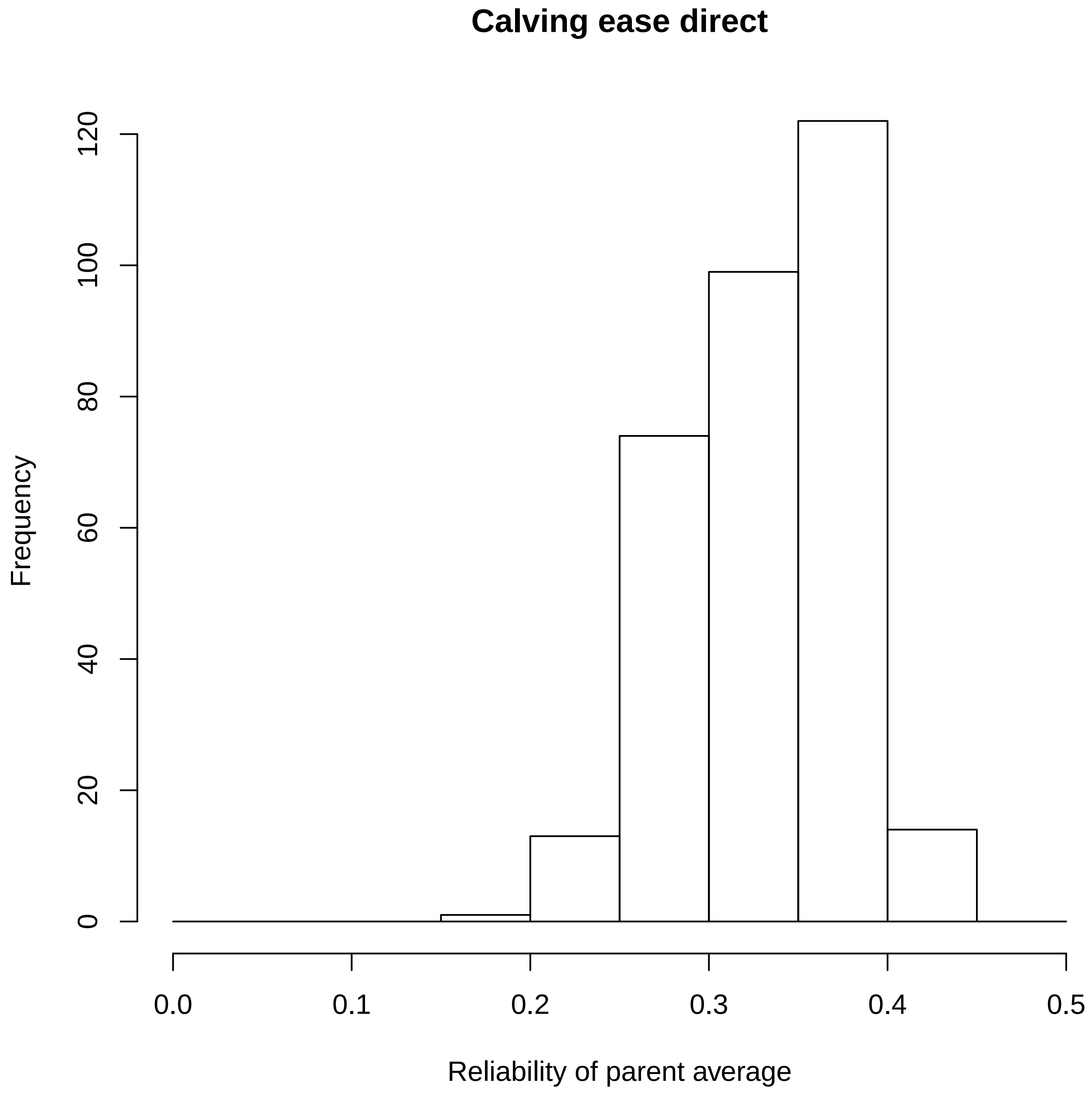
Calving ease direct
Frequency
0
20
40
60
80
100
120
0.0
0.1
0.2
0.3
0.4
0.5
Reliability of parent average

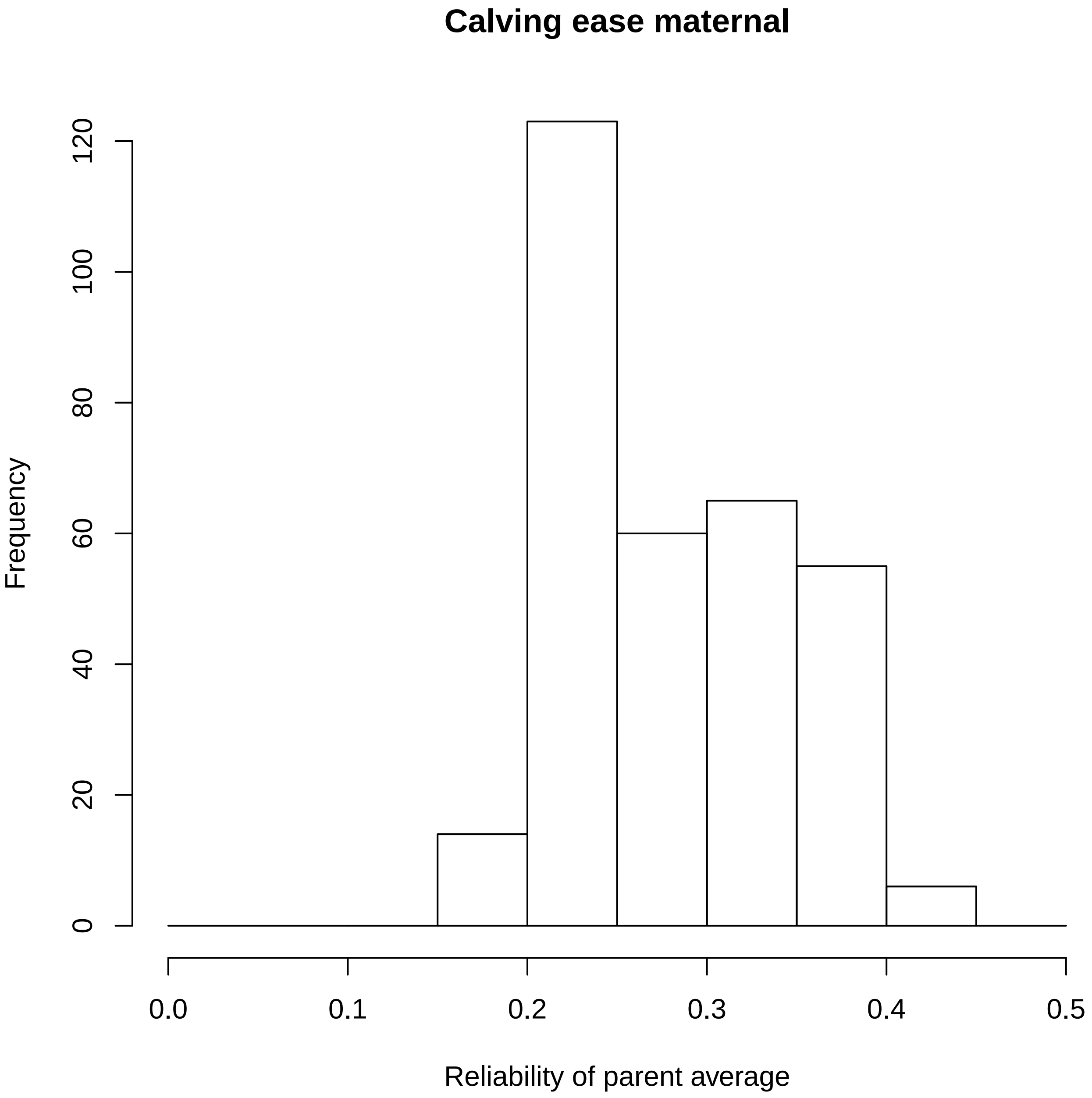

Calving ease maternal
Frequency
0
20
40
60
80
100
120
0.0
0.1
0.2
0.3
0.4
0.5
Reliability of parent average

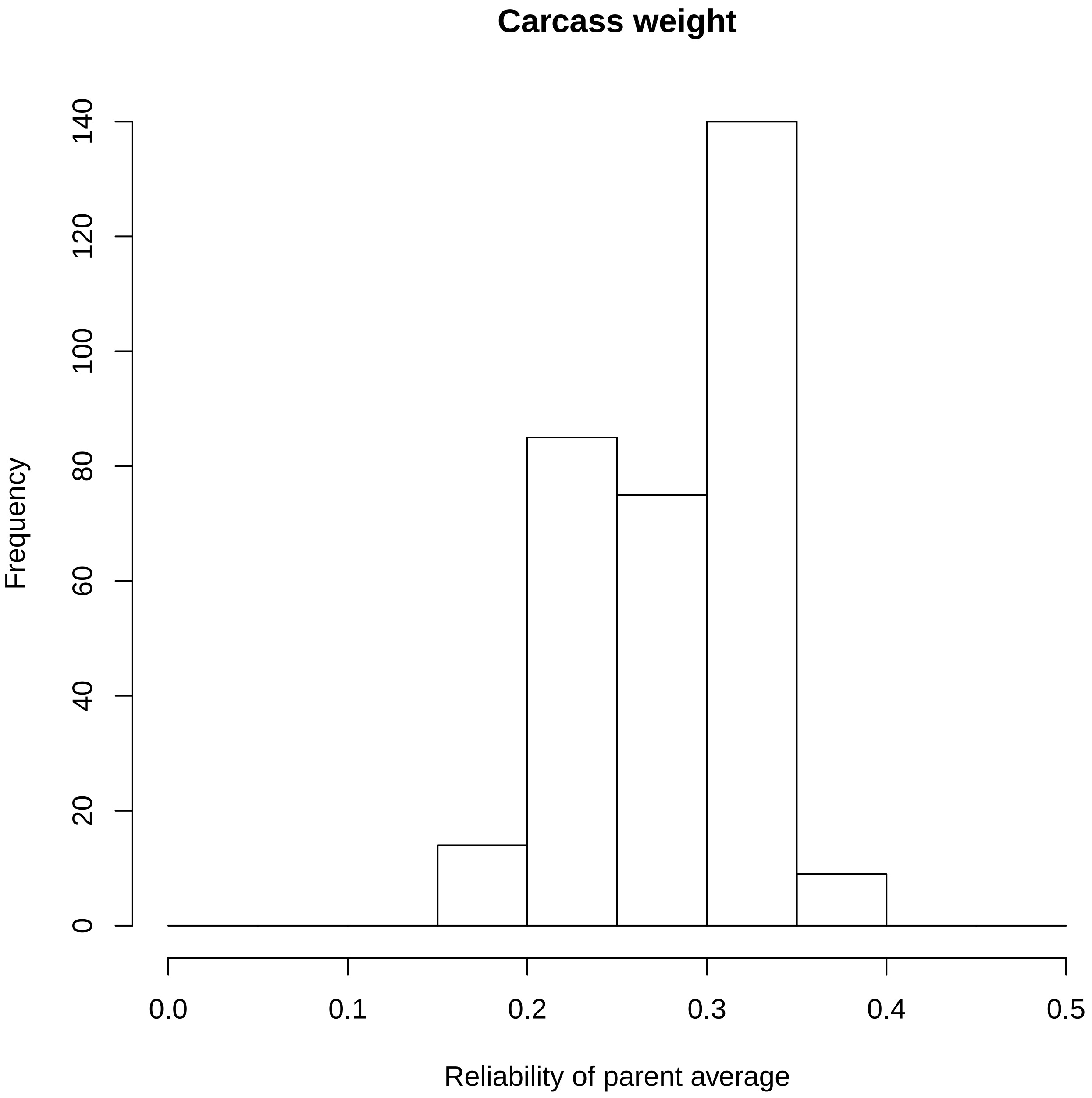
Carcass weight
Frequency
0
20
40
60
80
100
120
140
0.0
0.1
0.2
0.3
0.4
0.5
Reliability of parent average

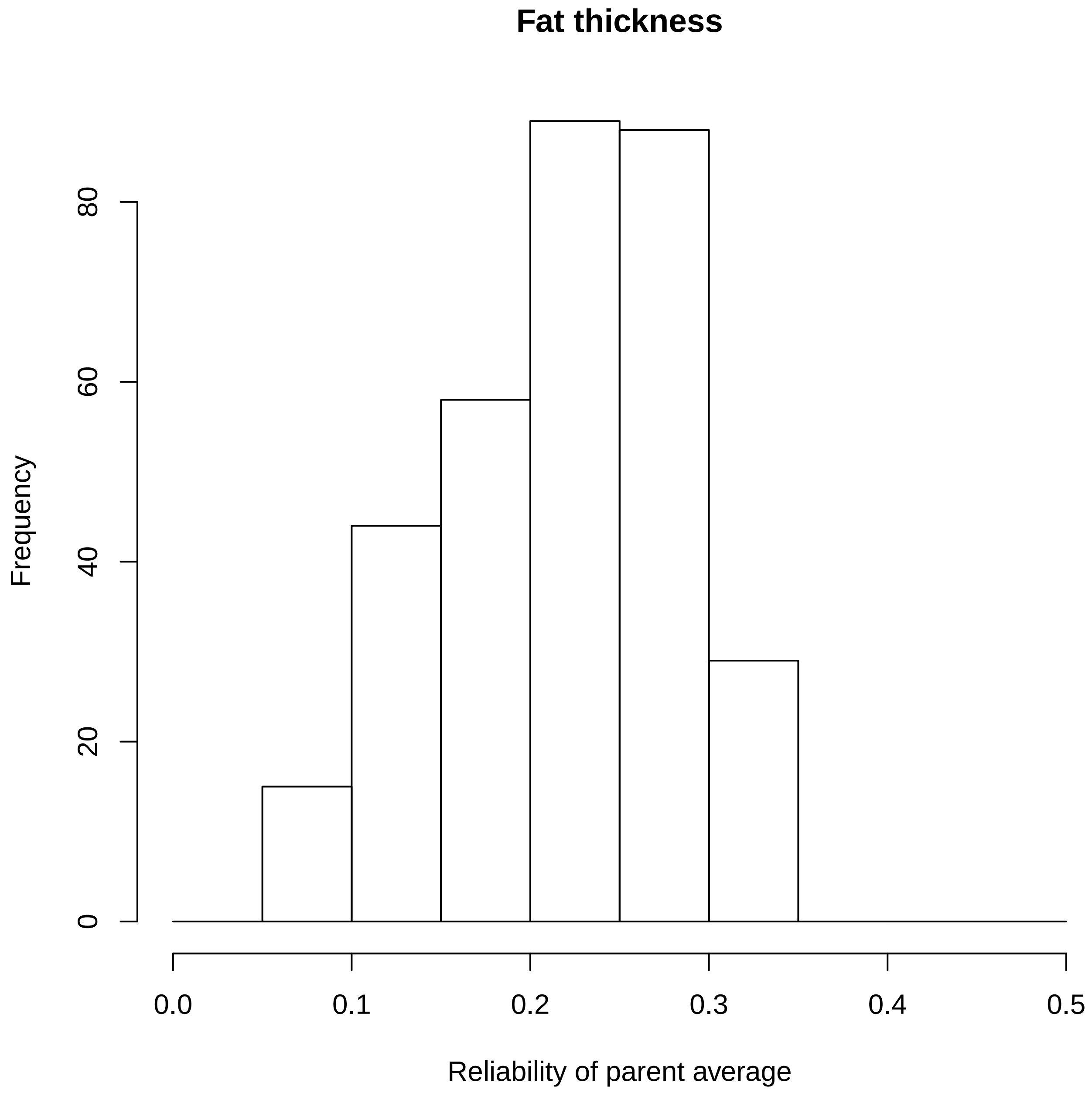

Fat thickness
Frequency
0
20
40
60
80
0.0
0.1
0.2
0.3
0.4
0.5
Reliability of parent average

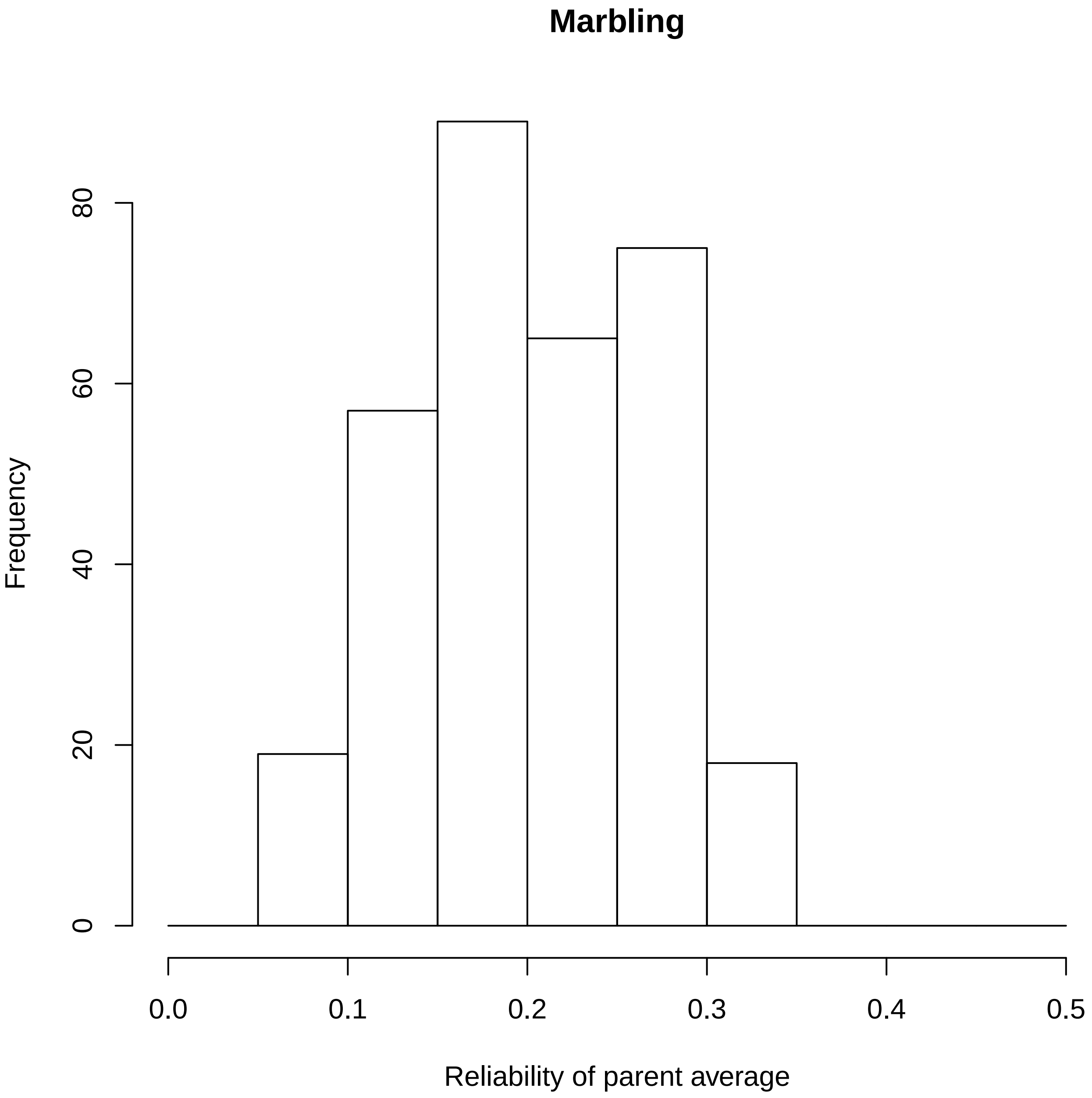

Marbling
Frequency
0
20
40
60
80
0.0
0.1
0.2
0.3
0.4
0.5
Reliability of parent average

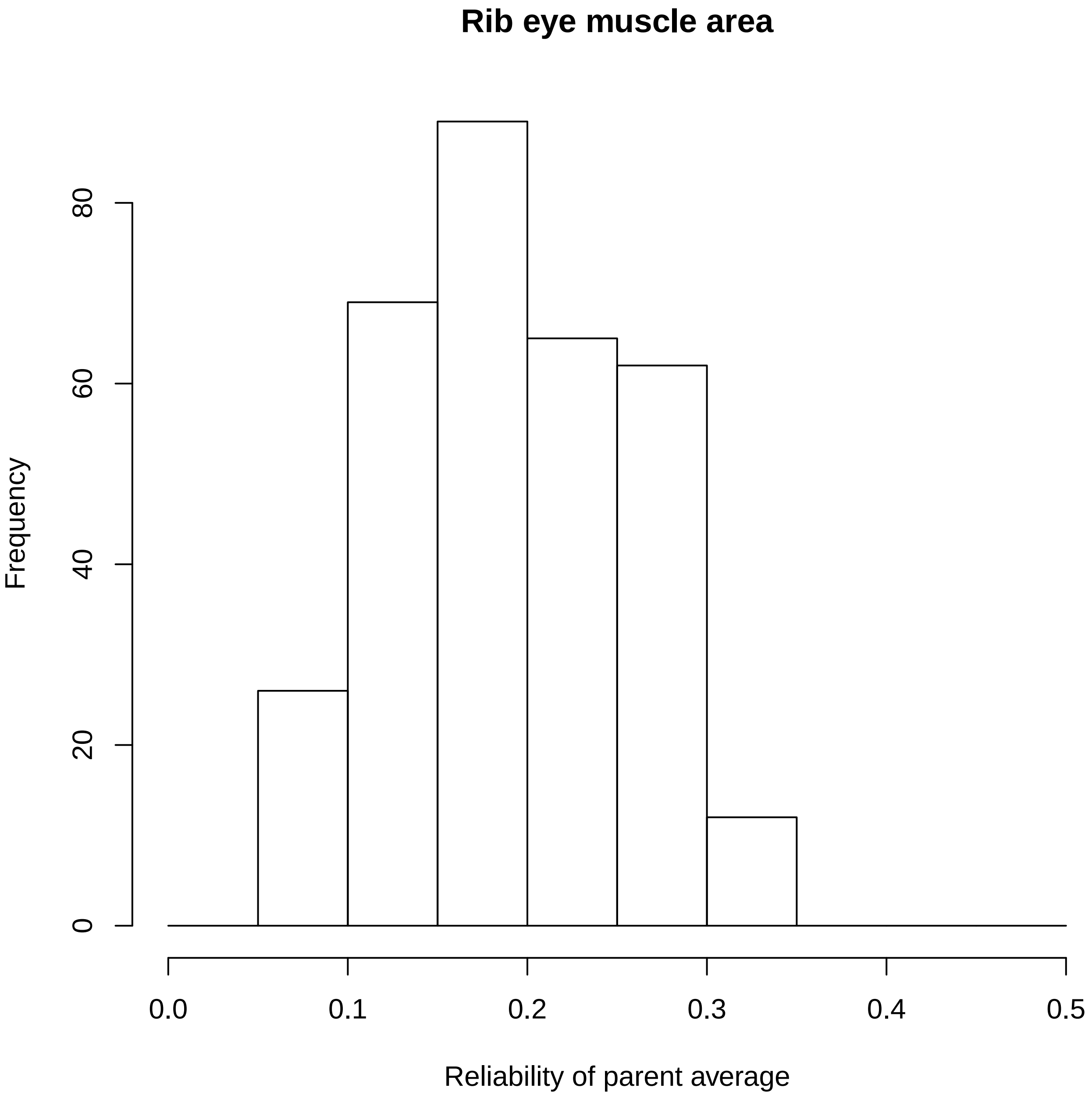

Rib eye muscle area
Frequency
0
20
40
60
80
0.0
0.1
0.2
0.3
0.4
0.5
Reliability of parent average

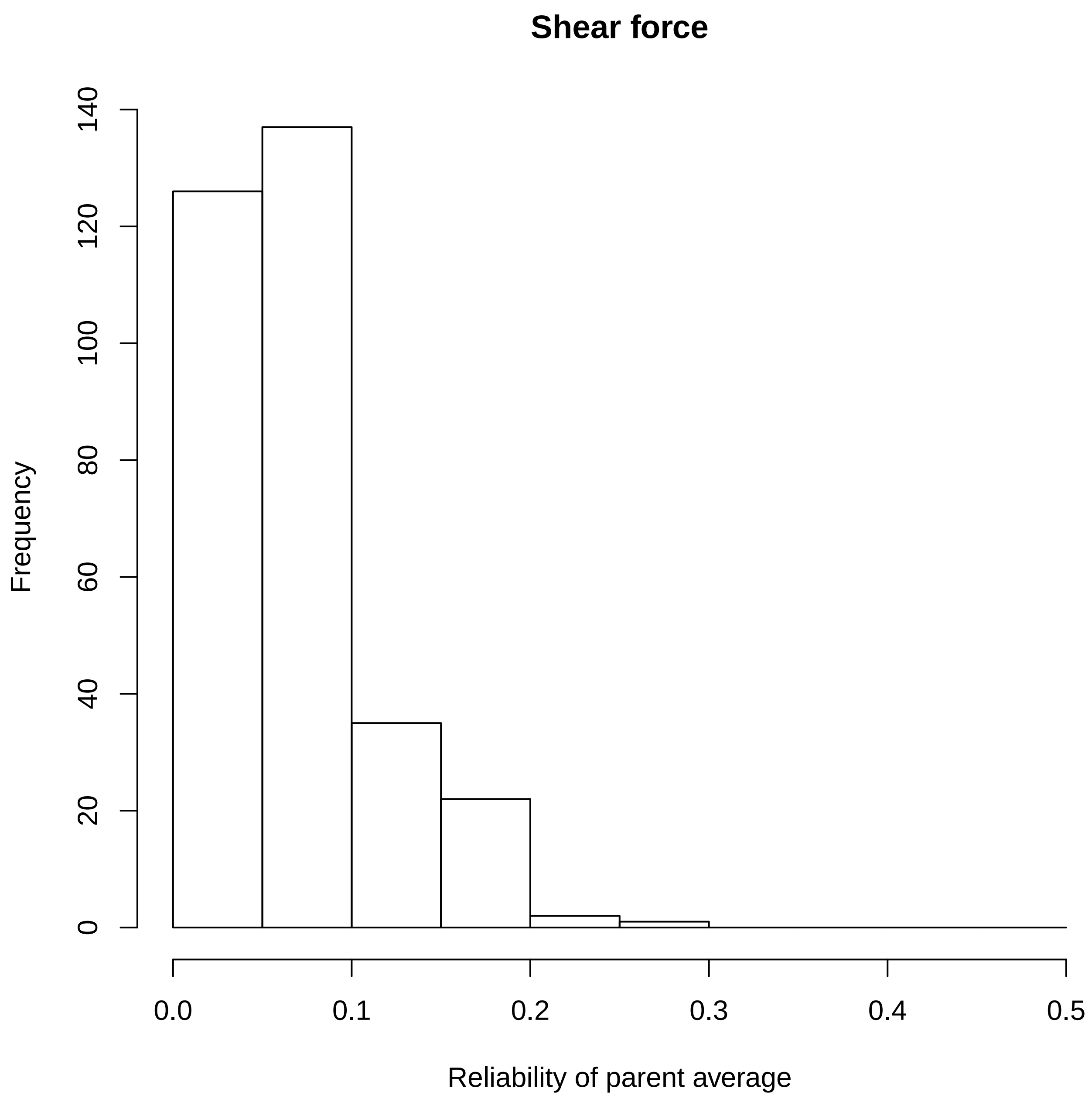

Shear force
Frequency
0
20
40
60
80
100
120
140
0.0
0.1
0.2
0.3
0.4
0.5
Reliability of parent average

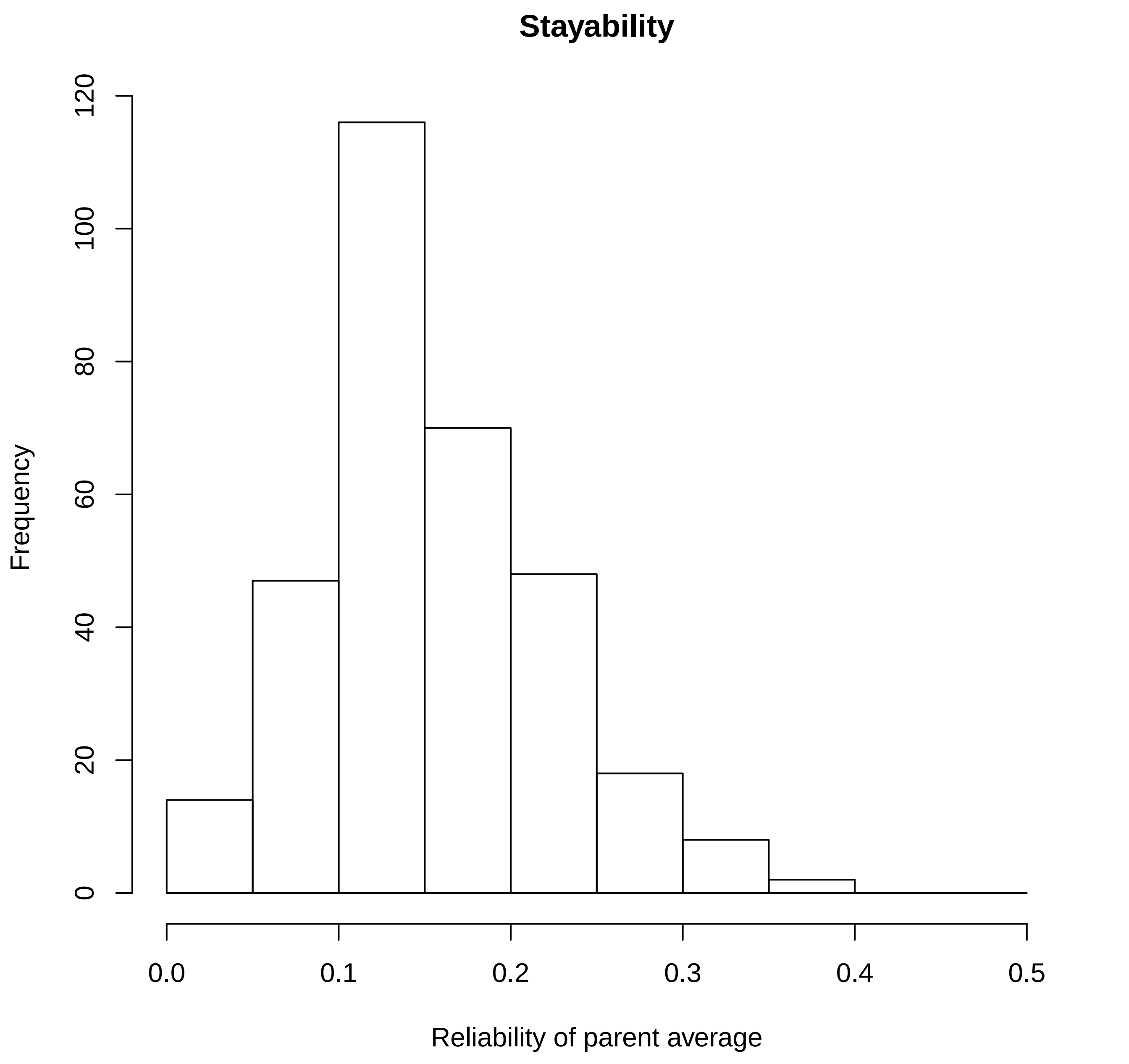
Stayability
Frequency
0
20
40
60
80
100
120
0.0
0.1
0.2
0.3
0.4
0.5
Reliability of parent average

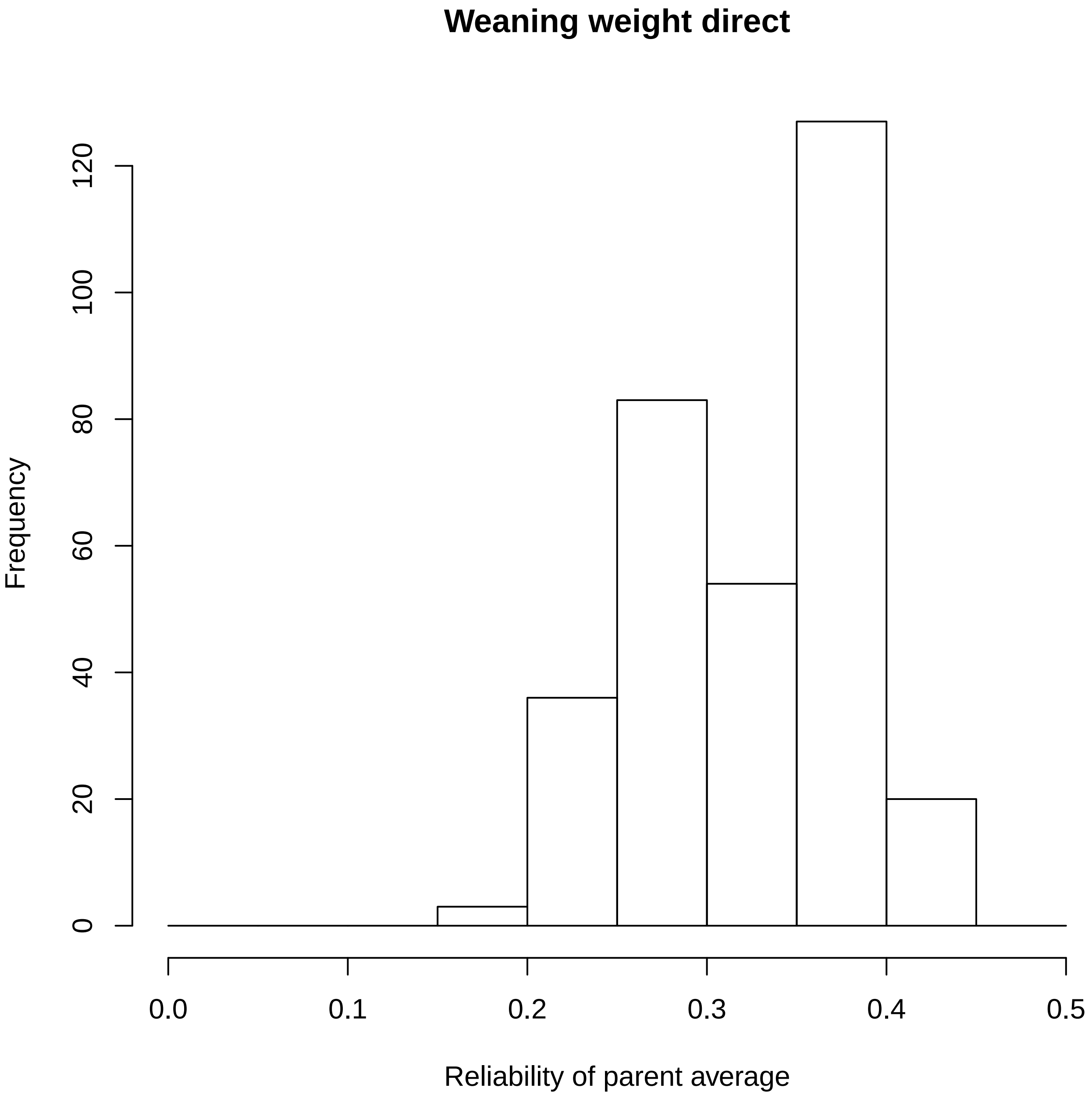
Weaning weight direct
Frequency
0
20
40
60
80
100
120
0.0
0.1
0.2
0.3
0.4
0.5
Reliability of parent average

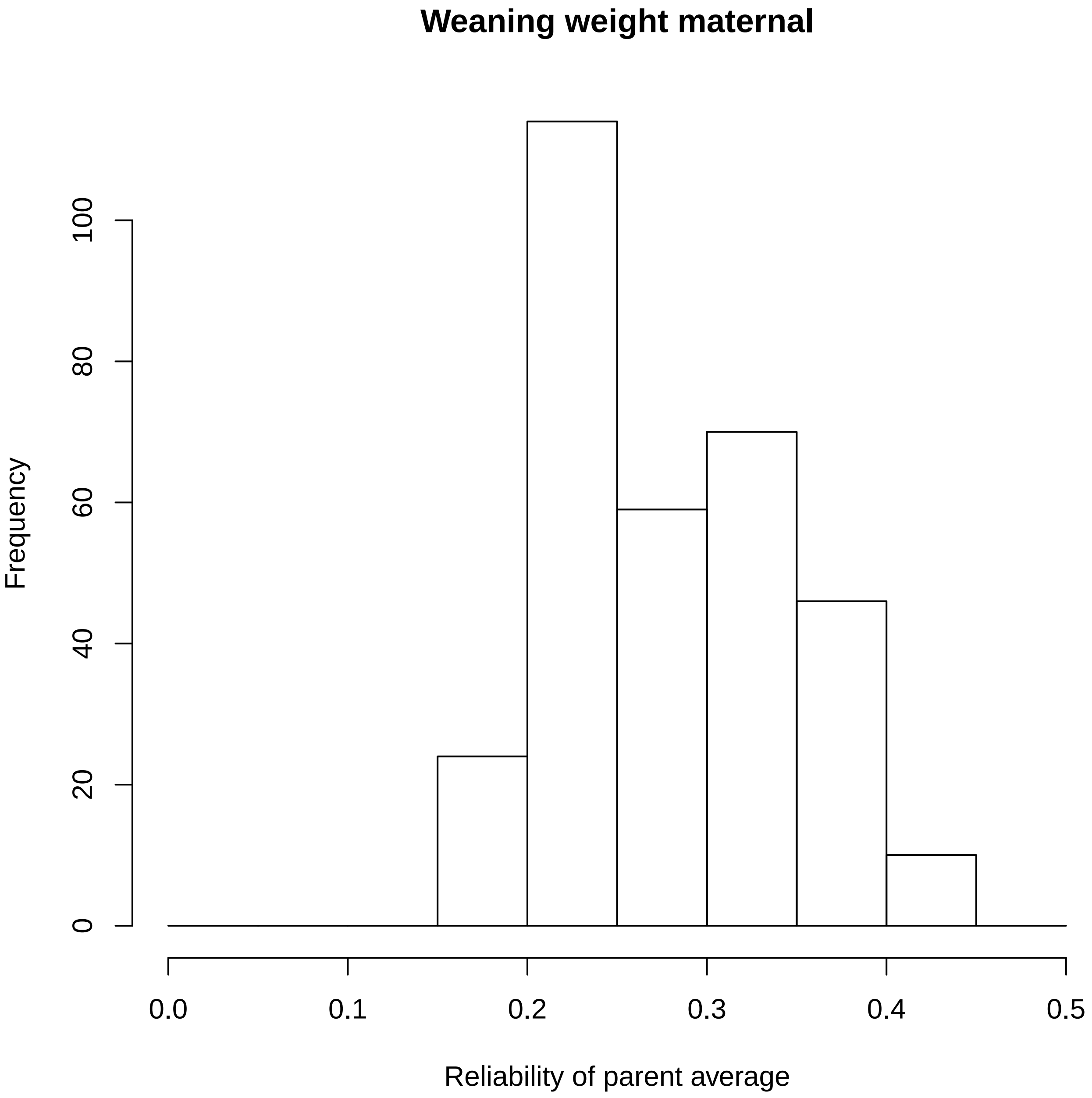

Weaning weight maternal
Frequency
0
20
40
60
80
100
0.0
0.1
0.2
0.3
0.4
0.5
Reliability of parent average

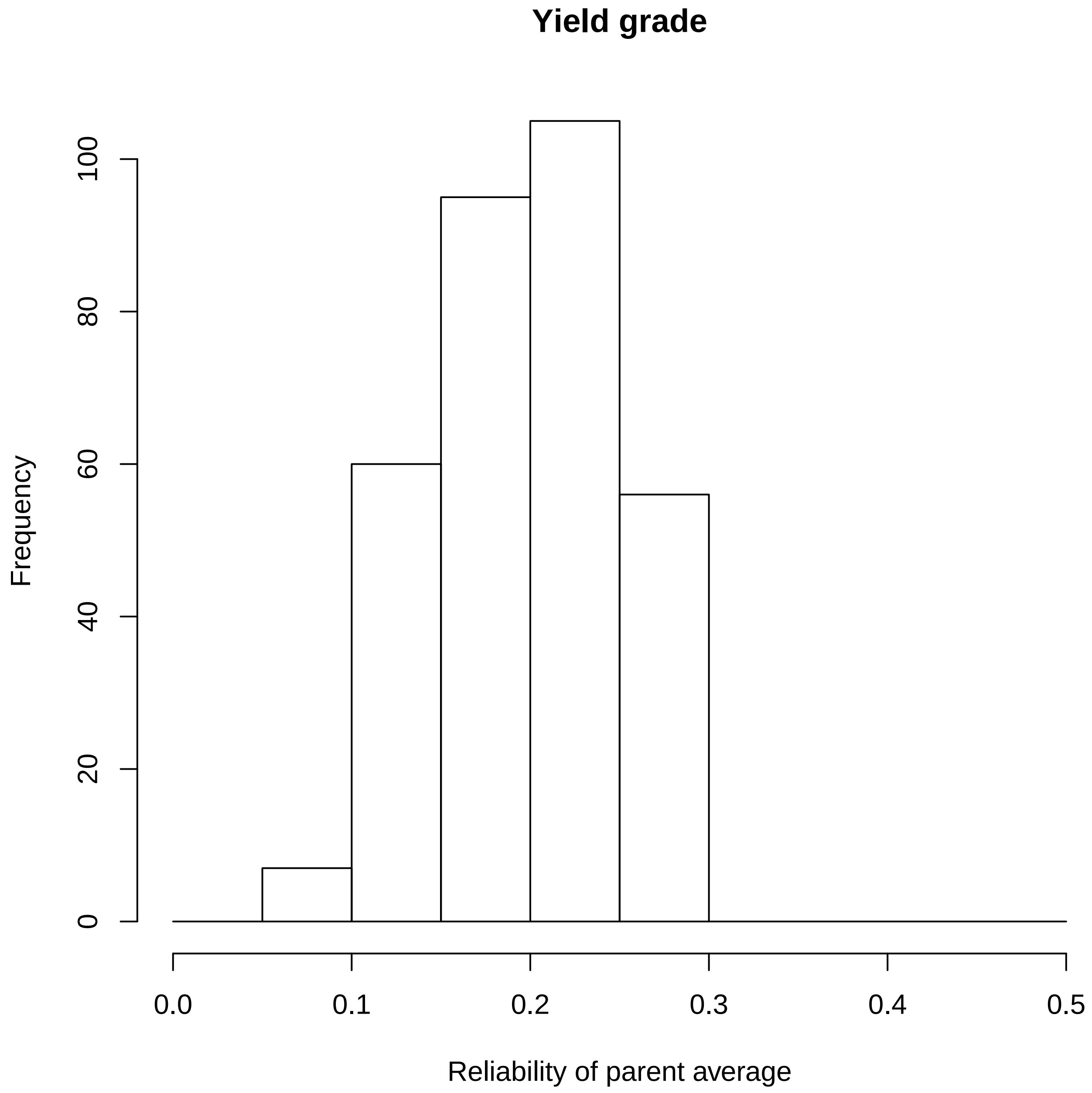

Yield grade
Frequency
0
20
40
60
80
100
0.0
0.1
0.2
0.3
0.4
0.5
Reliability of parent average

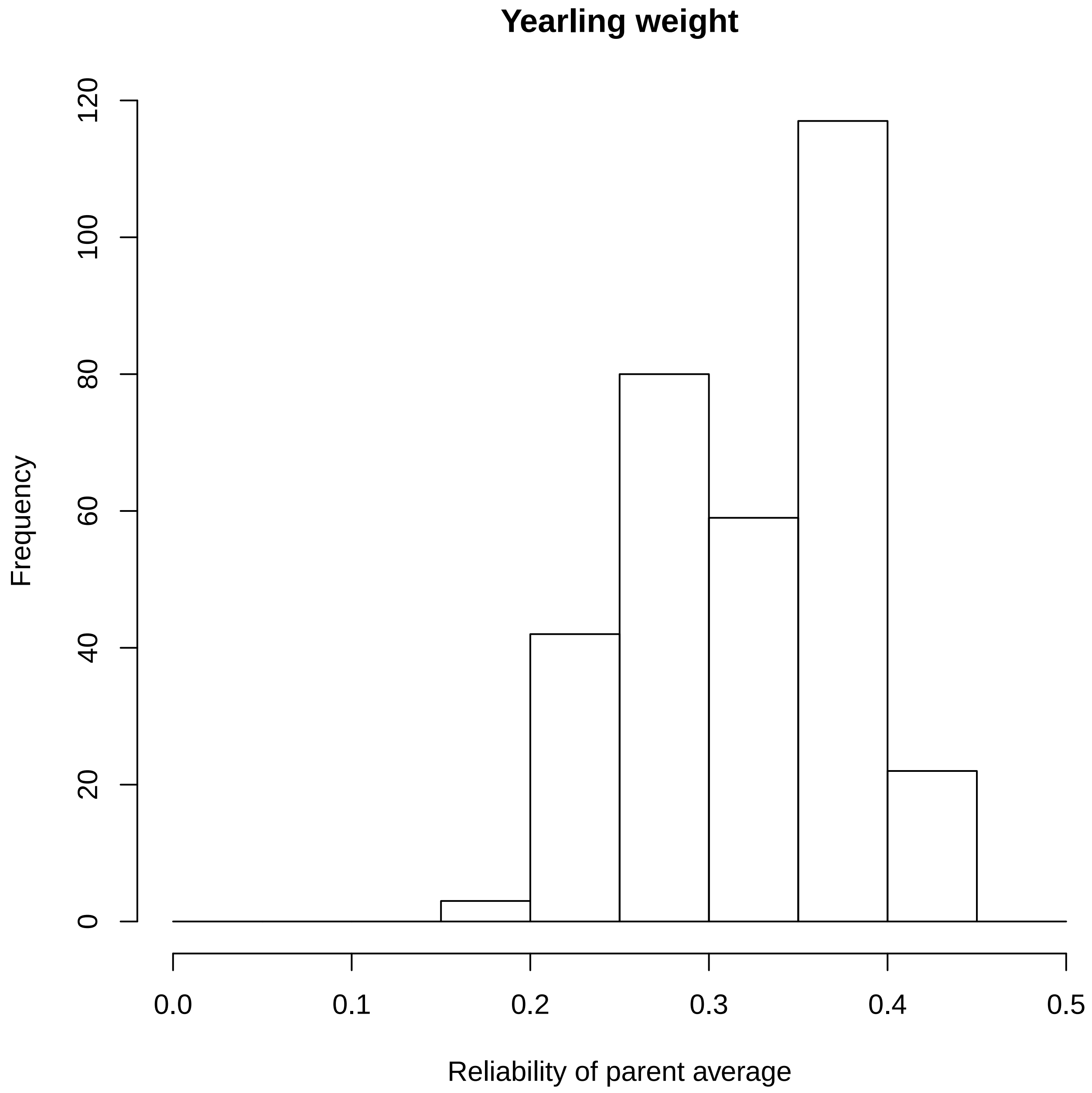
Yearling weight
Frequency
0
20
40
60
80
100
120
0.0
0.1
0.2
0.3
0.4
0.5
Reliability of parent average
